# Impacts of Satellite-Based Snow Albedo Assimilation on Offline and Coupled Land Surface Model Simulations

**DOI:** 10.1371/journal.pone.0137275

**Published:** 2015-09-14

**Authors:** Tao Wang, Shushi Peng, Gerhard Krinner, James Ryder, Yue Li, Sarah Dantec-Nédélec, Catherine Ottlé

**Affiliations:** 1 Laboratoire de Glaciologie et Géophysique de l’Environnement, UMR5183, CNRS/Université Joseph Fourier-Grenoble, Grenoble, France; 2 Key Laboratory of Alpine Ecology and Biodiversity, Institute of Tibetan Plateau Research, Chinese Academy of Sciences, Beijing, 100085, China; 3 Laboratoire des Sciences du Climat et de l’Environnement, CEA CNRS UVSQ, 91191, Gif-sur-Yvette, France; 4 Sino-French Institute for Earth System Science, College of Urban and Environmental Sciences, Peking University, Beijing, 100871, China; Universidade de Vigo, SPAIN

## Abstract

Seasonal snow cover in the Northern Hemisphere is the largest component of the terrestrial cryosphere and plays a major role in the climate system through strong positive feedbacks related to albedo. The snow-albedo feedback is invoked as an important cause for the polar amplification of ongoing and projected climate change, and its parameterization across models is an important source of uncertainty in climate simulations. Here, instead of developing a physical snow albedo scheme, we use a direct insertion approach to assimilate satellite-based surface albedo during the snow season (hereafter as snow albedo assimilation) into the land surface model ORCHIDEE (ORganizing Carbon and Hydrology In Dynamic EcosystEms) and assess the influences of such assimilation on offline and coupled simulations. Our results have shown that snow albedo assimilation in both ORCHIDEE and ORCHIDEE-LMDZ (a general circulation model of Laboratoire de Météorologie Dynamique) improve the simulation accuracy of mean seasonal (October throughout May) snow water equivalent over the region north of 40 degrees. The sensitivity of snow water equivalent to snow albedo assimilation is more pronounced in the coupled simulation than the offline simulation since the feedback of albedo on air temperature is allowed in ORCHIDEE-LMDZ. We have also shown that simulations of air temperature at 2 meters in ORCHIDEE-LMDZ due to snow albedo assimilation are significantly improved during the spring in particular over the eastern Siberia region. This is a result of the fact that high amounts of shortwave radiation during the spring can maximize its snow albedo feedback, which is also supported by the finding that the spatial sensitivity of temperature change to albedo change is much larger during the spring than during the autumn and winter. In addition, the radiative forcing at the top of the atmosphere induced by snow albedo assimilation during the spring is estimated to be -2.50 W m^-2^, the magnitude of which is almost comparable to that due to CO_2_ (2.83 W m^-2^) increases since 1750. Our results thus highlight the necessity of realistic representation of snow albedo in the model and demonstrate the use of satellite-based snow albedo to improve model behaviors, which opens new avenues for constraining snow albedo feedback in earth system models.

## Introduction

Snow albedo can exert a control over shortwave forcing in climate change and provides a strong feedback to the atmosphere, primarily because of the contrast in albedo between snow covered and snow free land surfaces [1, 2]. Snow albedo is also amongst the most important local parameters in the shaping of spatiotemporal variations in snowpack—solar radiation that is absorbed by the snowpack is one of the principal energy sources in its evolution. Our recent study reported that an increase of absorbed solar energy due to the reduction of snow albedo acted as an amplifying factor in shaping below-normal spring snow cover extent at high-latitude regions [3]. Therefore, snow albedo strongly influences the mass balance, and in particular the ablation, of the snowpack (e.g. [4]).

Snow-covered albedo is affected by many factors including snow grain size, solar zenith angle, liquid water content, snow impurities, layer structure in the snowpack, and snow depth [5–8]. Among these factors, snow grain size is the most important variable controlling snow-covered albedo. However, snow grain size is difficult to predict and is crudely parameterized in terms of the snow age and its temperature history (e.g. [9]). Thus, the parameterizations of snow-covered albedo are empirical in many land surface models (e.g. [10]), including the multi-layer snow module that has recently been developed in ORCHIDEE (ORganizing Carbon and Hydrology In Dynamic EcosystEms, [11]) [12]. Although physically based snow albedo models (e.g. [8], [13]) have emerged in recent years, they are not sufficiently validated in global applications. In addition to snow-covered albedo, the snow albedo scheme in the land surface model used for large-scale climate studies also needs a representation of snow cover fraction (SCF), the accuracy of which has been recognized as crucial in snow and climate simulations. For example, Wang and Zeng [14] evaluated snow albedo formulations among the four major weather forecasting and climate models and found that the bias in snow albedo simulation from three of models is mainly caused by their unrealistic SCF parameterizations. Previous studies (e.g. [15, 16]) have also identified that one of the largest uncertainties in modeling snow and its interactions with the atmosphere stems from SCF formulations at a grid cell scale in land surface-atmospheric models. Despite efforts in the development of SCF parameterizations in the past, the representation of SCF in land surface models including ORCHIDEE is still needed to be refined (e.g. [17]).

Our previous study at the site level has shown that the correction to the snow albedo scheme at exposed sites in ORCHIDEE could significantly improve snow simulations [12], but the bias in simulated albedo during the snow season after correction still exists. Snow albedo scheme in ORCHIDEE has an explicit treatment of the vegetation canopy by allowing snow albedo to vary with time [18], which definitely has strengths than some models using a fixed snow albedo over forests throughout the snow season (e.g. [19]). But this parameterization could still suffer from certain deficiencies since it does not consider the complex processes of canopy snow interception and unloading that may significantly impact snow albedo especially in boreal evergreen needleleaf forests [20]. This gives rise to the following question: to what extent can we expect improvements in snow simulations by further improving snow albedo simulation in ORCHIDEE? In addition, previous studies (e.g. [1]) have documented that the discrepancy in parametrizations of snow albedo (including both snow-covered albedo and SCF) across earth system models participating in the Coupled Model Intercomparison Project Phase 5 (CMIP5) leads to a large model spread of snow albedo feedback that is a source of uncertainty in climate simulations. Then, to what extent does realistic snow albedo representation improve air temperature due to the propagation of surface albedo changes to the atmosphere?

In this study, instead of developing a physical-based snow albedo scheme (e.g. [8]) in ORCHIDEE, we relax the modeled surface albedo during the snow season towards observations at each observed time step and quantify the changes in offline and coupled (land surface-atmosphere) simulations after this relaxation (or assimilation). To date, only a few examples exist of the assimilation of albedo into land surface models on the continental scale (e.g. [21]); let alone exploring impacts of snow albedo assimilation in the coupled land-atmosphere models. This is primarily because most albedo products have large spatial and temporal gaps that hamper their assimilation into such models. Our snow albedo assimilation framework not only uses in-situ albedo measurements at the site level but also capitalizes on a newly released global-covered albedo product (GlobAlbedo, [22]) with no data gap at the continental level. Use of this newly released snow albedo product and a recently developed ORCHIDEE snow module could enable us to quantify the simulation errors owing to model deficiency in snow albedo representation. The primary purpose of this paper is thus to demonstrate the importance of appropriately representing surface albedo during the snow season in both ORCHIDEE and the coupled ORCHIDEE-LMDZ (a general circulation model of Laboratoire de Météorologie Dynamique) model.

## Materials and Methods

### Study sites

We use two sites (Weissfluhjoch and Col De Porte) to investigate the impacts of snow albedo assimilation on simulated snow depth and upward shortwave radiation in ORCHIDEE. Weissfluhjoch (WFJ, 1992–1993) is a high-elevation site at 2540 m with flat topography, located in the eastern Swiss Alps (46.83°N, 9.81°E) and managed by the Swiss Federal Institute for Snow and Avalanche Research. The average air temperature during the period of continuous snow cover is -2.9°C. Rainfall does not occur from mid-October to mid-May. Snow continuously accumulates from mid-October until mid-April and then melts through May and June owing to temperatures above the melting temperature. On this site, the meteorological forcing used for driving ORCHIDEE is provided. Evaluation data comprise hourly observations of snow depth from an ultrasonic sensor and daily snow albedo. Data from this site have been used in the assessment of many snow models (e.g. [23–25]).

The Col De Porte (CDP, 1997–1998) experimental site (1325 m altitude, 45.8°N, 5.75°E) is situated in the Chartreuse mountain range near Grenoble, France. This site is located in a grassy meadow surrounded by a coniferous forest. The snow often begins in November and ends at the beginning of May. Winter air temperature can occur intermittently above the freezing point throughout the winter, and rainfall episodes can thus be common during the snow season. The soil generally does not freeze. Besides hourly observations of meteorological data used for driving ORCHIDEE, hourly snow depth from an ultrasonic sensor and daily snow albedo are also provided. This site has been widely used to evaluate snow schemes (e.g. [12], [24–27]).

### Large-scale datasets

#### Climate forcing

The meteorological forcing CRUNCEP is used to drive ORCHIDEE in an offline mode. CRUNCEP is a merged product of the Climate Research Unit (CRU) monthly 0.5° climatology (v5.3.2, 1901–2012) observations and the NCEP (National Centers for Environmental Prediction) reanalysis data with a high temporal resolution. This merged product has a six-hourly temporal and 2° spatial resolution. In addition, climatological variables (e.g. air temperature at 2 m and incoming shortwave radiation) in CRUNCEP are also used for evaluating the coupled ORCHIDEE-LMDZ simulations.

#### GlobAlbedo product

The GlobAlbedo product is generated by employing multiple sensors (SPOT4-VEGETATION, SPOT5-VEGETATION2 and MERIS) [22]. Both black-sky (Direct Hemispherical Reflectance) and white-sky (BiHemispherical Reflectance) albedo in three spectral broadband ranges (shortwave: 400–3000 nm, visible: 400–700 nm and near- and shortwave-infrared: 700–3000 nm) are provided. A detailed description of the GlobAlbedo processing system can be found at: (http://www.globalbedo.org/). To achieve assimilation of surface albedo during the snow season into the land surface model ORCHIDEE, we need the blue-sky albedo (*α*_*blue*_) that refers to the instantaneous surface albedo measured under natural daylight illumination. *α*_*blue*_ can be approximately expressed as a linear combination of black (*α*_*black*_) and white-sky albedo (*α*_*white*_) [28, 29].

$$\alpha_{blue}=(1-D)\alpha_{black}+D\alpha_{white}$$ (1)

**Fig 1:**
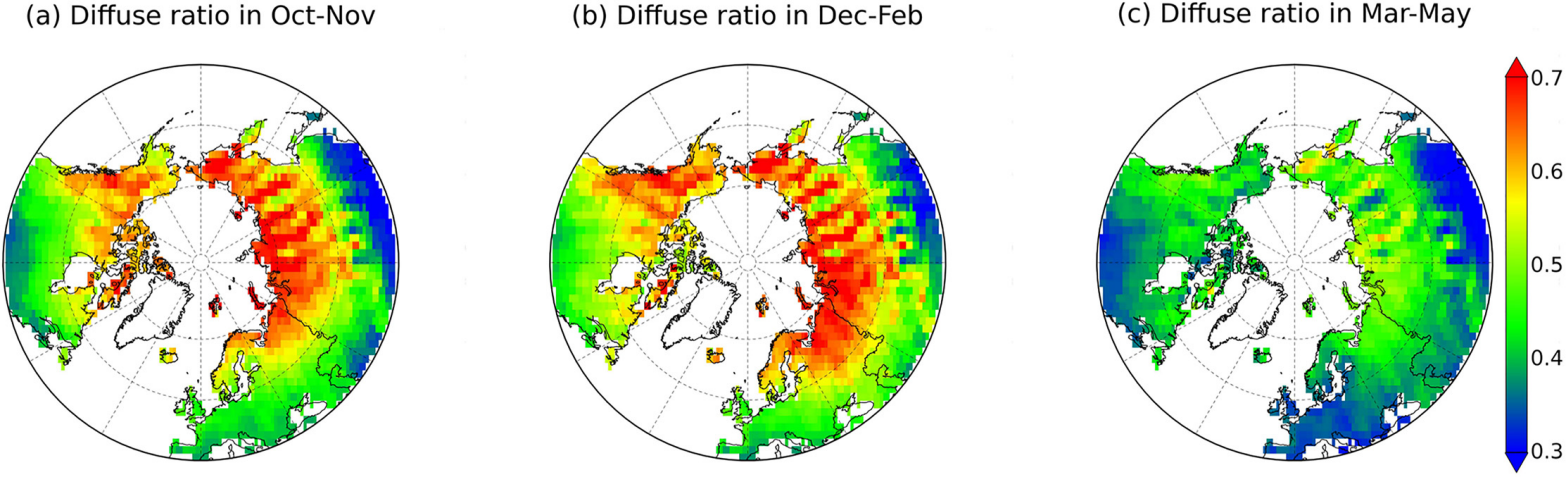
Mean diffuse skylight ratio derived from NCEP data for autumn (October and November), winter (December, January and February) and spring (March, April and May) seasons over the period of 1998–2011.

Where *D* denotes the fraction of diffuse skylight. We use 8-day diffuse and direct downward radiation data from the National Centers for Environmental Prediction (NCEP) reanalysis to obtain *D* (e.g. [30]). Fig 1 shows mean *D* during the autumn (October and November), winter (December, January and February) and spring (March, April and May) over the period 1998–2011. The computed blue-sky albedo in the shortwave range with an 8-day period and a spatial resolution of 0.5 degrees is then used for snow albedo assimilation in ORCHIDEE. In addition, SCF from GlobAlbedo product is also used. Both blue-sky albedo and SCF are resampled to 2° x 2° for offline model runs and 3.75° x 2.5° for coupled model runs. The albedo refers to blue-sky albedo in the following text.

#### GlobSnow SWE product

The monthly snow water equivalent (SWE) from GlobSnow [31]) is adopted to evaluate mean seasonal (October throughout May) SWE over the region north of 40 degrees. GlobSnow SWE product is derived from a combination of ground based data and satellite microwave radiometer-based measurements (SMMR, SSM/I and AMSR-E sensors) from 1979 until present [31]. The GlobSnow SWE processing system uses passive microwave observations and weather station observations in an assimilation scheme to produce maps of SWE estimates over the Northern Hemisphere. The complete algorithm evaluation, including an overview of the algorithms, reference datasets, and the results are presented in the GlobSnow design justification file [31]. The product with mountainous regions unmasked is used in this study. Previous validation of the GlobSnow SWE retrievals demonstrated RMSE values of 30 to 40 mm for SWE values below 150 mm and acknowledged that further improvement is necessary to better account for land cover and forest properties and the effect of lakes [31].

### Snow albedo assimilation in ORCHIDEE

#### The default snow albedo scheme

In the absence of fresh snow, snow-covered albedo decreases exponentially with time from its fresh value to an old value that is specific for each plant function type (PFT) in ORCHIDEE. The snow-covered albedo for each PFT is parameterized using the following equation,

$$\alpha_{snow}^{jv}=A_{old}^{jv}+B_{decay}^{jv}\text{exp}(-\frac{sage}{5})$$ (2)

Where $\alpha_{snow}^{jv}$ denotes snow-covered albedo for each PFT *jv*, $A_{old}^{jv}$ and $B_{decay}^{jv}$ denote minimum snow-covered albedo after aging (old snow) and decay rate of snow-covered albedo for each PFT, respectively (Table 1). $A_{old}^{jv}+B_{decay}^{jv}$ corresponds to albedo measured for fresh snow. The *sage* (in days) represents snow age that is parameterized as,

$$sage(t+1)=(sage(t)+(1-\frac{sage(t)}{50})\times \frac{dt}{86400})\times \text{exp}(-\frac{snowf(t)}{0.3})$$ (3)

*dt* (1800s) is the model time step that is expressed in days. *snowf* is defined as snowfall amount (m) during *dt*. Snow age *sage* is updated at each model time step *dt*, and decreases with *snowf*.

**Table 1:** Default snow albedo parameters for different plant function types in ORCHIDEE.

Plant function type	Old snow (*A*_*old*_)	Decay rate (*B*_*decay*_)
Bare soil/grass/crop	0.55	0.30
Temperate needleleaf evergreen	0.14	0.06
Temperate broad-leaved evergreen	0.15	0.14
Temperate broad-leaved summergreen	0.15	0.14
boreal needleleaf evergreen	0.14	0.06
boreal broad-leaved summergreen	0.15	0.25
boreal needleleaf summergreen	0.14	0.06

The snow-covered albedo at the grid scale (*α*_*snow*_) is then computed as the sum of PFT-specific snow-covered albedo, weighted by the PFT fraction *f*_*jv*_.

$$\alpha_{\text{snow}}=\sum_{jv=1}^{}f_{jv}\alpha_{snow}^{jv}$$ (4)

The SCF on each grid box is a function of snow depth (*SD*) and snow density (*SRHO*), as given by,

$$SCF=\text{tanh}(\frac{SD}{0.025\times \frac{SRHO}{50}})$$ (5)

The total surface albedo at the grid scale is then computed as the sum of snow-free albedo (*α*_*ns*_) and snow-covered albedo (*α*_*snow*_), weighted by SCF.

$$\alpha_{surf}=SCF\times \alpha_{snow}+(\text{1}-SCF)\times \alpha_{ns}$$ (6)

The surface albedo is used to compute the absorbed shortwave radiation that is an important radiative flux in surface energy budget, from which skin temperature over the snow surface can be resolved in ORCHIDEE. The impacts of albedo on snow dynamics (snow accumulation and ablation) are then manifested through changing the snow temperature profile, which is updated from the skin temperature at each model time step using backward-difference implicit integration scheme of ORCHIDEE.

#### Snow albedo assimilation technique

In this study, we use a direct insertion data assimilation approach. This relatively simple method differs from more advanced techniques (such as the Kalman filter) which statistically combine model forecasts with observations based on their uncertainties (e.g. [32, 33]). In default albedo scheme of ORCHIDEE, several PFTs can exist in one grid cell. If each PFT has one parameter used for tuning (e.g. old snow albedo *A*_*old*_), more than one parameter should then be tuned to match observed surface albedo for grid cells with several PFTs co-exist. This is almost impossible for us since a relatively simple assimilation technique will be used in this study. Thus, we replace the default snow albedo scheme in ORCHIDEE (Eqs 2 and 4) with the one that is adapted from the U.S. Army Corps of Engineers [34], and which has already been implemented in many land surface models (e.g. [35, 36]). In this scheme, the snow-covered albedo *α*_*snow*_ at the grid scale is parameterized as:

$$\alpha_{snow}=\alpha_{tune}\times A^{sage^{B}}$$ (7)

Where the two decay parameters A and B are prescribed as global constants. The values of two decay parameters defined differently for dry and wet snowpack. The parameters A and B are equal to 0.94 and 0.58 (0.82 and 0.46), respectively, during accumulation (melt) phase. The criterion for decay is simulated skin temperature from ORCHIDEE reaches 273.15 K, such that when it is below (at) freezing, the albedo is computed using the accumulation (melt) decay rate. *sage* (in days) representing snow age is computed based on Eq 3. *α*_*tune*_ is recognized as the albedo value of fresh snow in the original formulation [34], and its value is set to be 0.85.

To match the modeled surface albedo to observed albedo during the snow season, modeled SCF is directly replaced with GlobAlbedo SCF and *α*_*tune*_ (related to snow-covered albedo) then becomes the only tunable parameter (e.g. [37]) in the following Eq 8. We do not tune snow-free albedo (*α*_*ns*_) in Eq 8 given that the magnitude of snow-free albedo is always smaller than snow-covered albedo. The variation of surface albedo during the snow season can then be mainly attributed to changes in snow-covered albedo.

$$\alpha_{surf}=SCF\times \alpha_{tune}\times A^{sage^{B}}+(\text{1}-SCF)\times \alpha_{ns}$$ (8)

Since ORCHIDEE simulates albedo on a half-hourly timescale, *α*_*tune*_ is tuned only at the first model time-step of each 1-day period (or 8-day period) to match the corresponding surface albedo during the snow season from in-situ measurements (or satellite products) over the same period. There are 48 (or 384) model time-steps within each 1-day (or 8-day) period. *α*_*tune*_ would thus be maintained over the course of the remaining time-steps within either of these time periods. This snow albedo update from the observations does not affect the decay rates and the integrity of the albedo physics is preserved. Note that the modeled SCF will be replaced with GlobAlbedo SCF at each model time step (*dt* = 1800s) in Eq 8. Since GlobAlbedo SCF is reported at the time scale of 8 days, the linear interpolation between 8-day values is simply adopted to obtain the SCF at each model time step. This simple linear interpolation may introduce the bias especially during the late snowmelt season since the decrease of spring snow cover can be non-linear, which highlights the use of high temporal resolution dataset (e.g. MODIS daily SCF) in the future study. To be consistent with the albedo data, we still use 8-day GlobAlbedo SCF in this study.

At the continental scale, assimilation of surface albedo only occurs when GlobAlbedo SCF is larger than 10%. Although albedo from GlobAlbedo product was used for assimilation, it is also employed for evaluating the success degree of snow albedo assimilation in ORCHIDEE since GlobAlbedo has a much coarser time resolution (8-day period) than the model time step (30 min).

### Experimental design

#### Offline experiments

At the site level, in order to explore the impact of the frequency of albedo observations on modeled results, we compare simulations that were assimilated with daily in-situ albedo (hereafter ASS1) against those using albedo sampled over each 8 day period that was derived from an average of daily in-situ albedo (hereafter ASS8). All site simulations are carried out by setting a snow fraction of 1, which is recommended for local scale applications with an emphasis on studying snow physics (e.g. [12, 27]). In addition, this is also considered because SCF is not available at the site level.

At the continental scale, we perform CTRL (an open-loop simulation based on the default snow albedo scheme) and ASSALB (a simulation that assimilates SCF and albedo based on the snow albedo scheme adapted from the U.S. Army Corps of Engineers) experiments from January 1998 to December 2011 in the region north of 40 degrees. In addition, in order to quantify the contribution of importing GlobAlbedo SCF to snow albedo simulation in ORCHIDEE, we also carry out an additional experiment called ASSSCF that only assimilates GlobAlbedo SCF using the default snow albedo scheme. That’s to say, we use the default snow albedo scheme in ORCHIDEE to simulate the snow-covered albedo for each grid cell (Eqs 2–4) and replace the simulated SCF with GlobAlbedo SCF at each model time step (*dt* = 1800s) in Eq 6. We use the default snow albedo scheme in ASSSCF given that the albedo scheme used in snow albedo assimilation has the same parameter (*α*_*tune*_) independent of PFTs, which is less realistic than the default snow albedo scheme having PFT-specific parameter values (Table 1).

#### Coupled experiments

We use ORCHIDEE-LMDZ to quantify impacts of snow albedo assimilation on air temperature and SWE. LMDZ is a global atmospheric general circulation model designed for climate studies, and the coupling between LMDZ and ORCHIDEE is described in Hourdin et al. [38]. We performed two simulations using a uniform horizontal resolution of 3.75 in longitude and 2.5 in latitude and 19 vertical layers. The first simulation, referred to as CTRL-COU, is the control experiment. The second simulation, referred to as ASSALB-COU, is identical to the control, except that it assimilates SCF and surface albedo from GlobAlbedo. Multi-year (1998–2011) mean SCF and surface albedo from GlobAlbedo are used in ASSALB-COU. All experiments are driven by 14-year (1998–2011) climatological monthly mean sea surface temperature and sea ice concentration with a spatial resolution of 1 degree taken from PCMDI (Program for Climate Model Diagnosis and Intercomparison). Each simulation is conducted for 19 years with the first 5 years as spin-up.

## Results and Discussion

### At the site level

**Fig 2:**
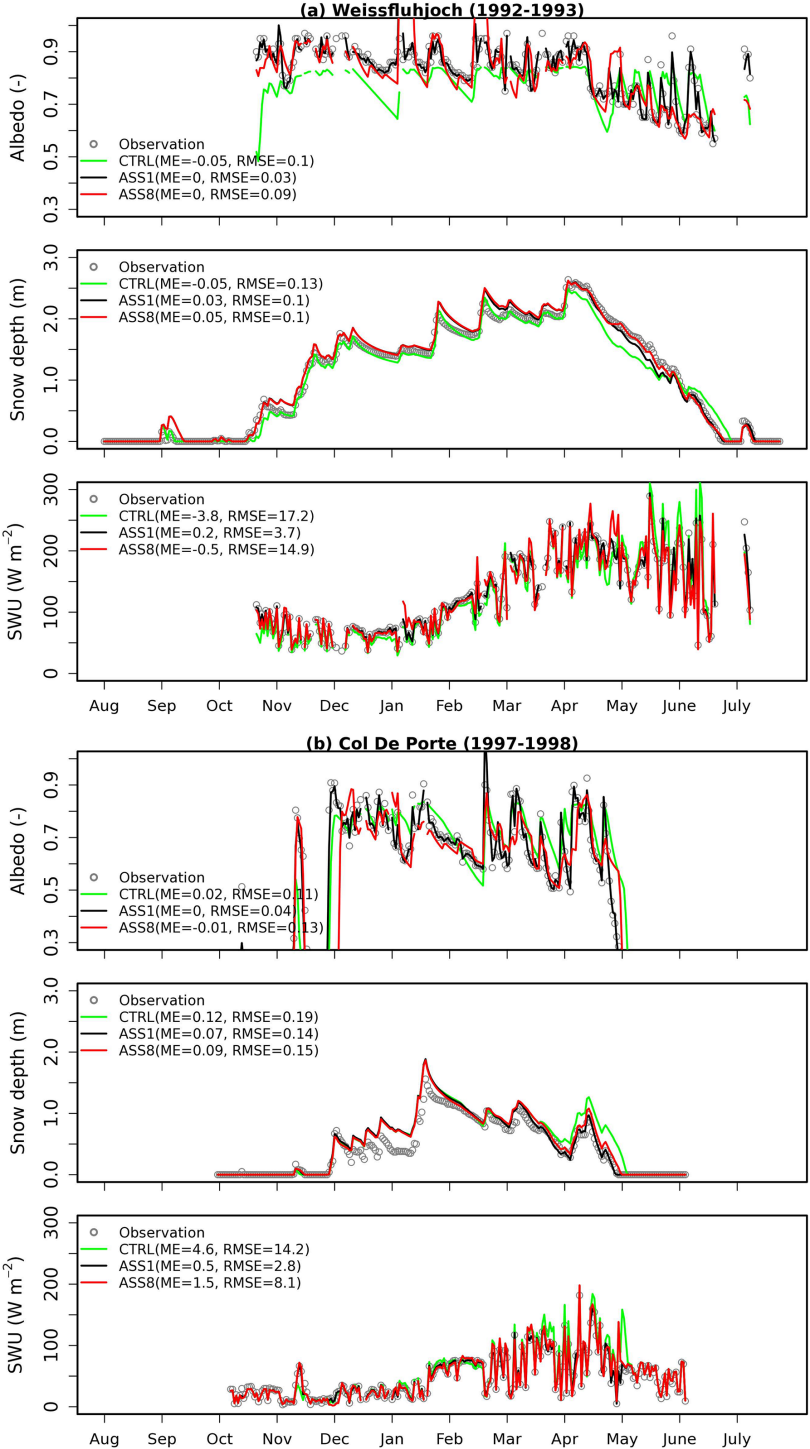
Comparisons of albedo, snow depth (m) and upward shortwave radiation (SWU) among observation, CTRL, ASS1 and ASS8 on both Weissfluhjoch (a) and Col De Porte (b) sites. CTRL denotes the ORCHIDEE control simulation without snow albedo assimilation. ASS1 and ASS8 represent ORCHIDEE simulations assimilated with daily albedo and albedo sampled on each 8-day period, respectively. ME and RMSE denote mean error and root mean square error respectively.

Fig 2 shows that simulated daily albedo from ORCHIDEE becomes close to the observations in both ASS1 and ASS8, which suggest that snow albedo assimilation into ORCHIDEE has been successful on both WFJ and CDP sites. For example, mean error (ME) approaches zero for both ASS1 and ASS8. The simulated daily albedo from ASS1 has a much smaller root mean square error (RMSE) than that from ASS8 (Fig 2), which indicates that higher temporal resolution of the assimilated albedo is required, in order to achieve improved simulation accuracy. But we do still observe the difference between observations and simulated albedo from ASS1. This is due to the fact that snow albedo in ORCHIDEE is simulated on a half-hourly timescale and only the first model time step within a day (that is composed of 48 model time-steps) is relaxed towards the in-situ measured albedo of that day. We do not assess the potential utility of the albedo that is extracted from GlobAlbedo with a temporal resolution of eight days. This is predominantly because the albedo obtained from GlobAlbedo, of a relatively low spatial resolution (5 km^2^), could have difficulty in representing accurately the in-situ measured albedo at the site level.

Fig 2 also displays the comparison between observed and simulated snow depth from CTRL, ASS1 and ASS8 on both sites. The multi-layer snow module in ORCHIDEE [12] generally performs well in CTRL, so there is not much room for improvement in snow depth simulations assimilated with in-situ measured albedos in terms of error statistics. If we take a closer look at the simulations during the snowmelt period, CTRL significantly underestimates the snow depth in the period of April to May at both sites, a bias that does not exist in either ASS1 or ASS8. This implies that a correct snow albedo appears to be more important during the snow ablation period. Furthermore, the decrease in RMSE (W m^-2^) after albedo assimilation may be clearly observed in upward shortwave radiation, especially in ASS1 (Fig 2). For example, the RMSE decreases from 17.2 to 3.7 W m^-2^ on WFJ site (Fig 2a) and from 14.2 to 2.8 W m^-2^ on CDP site (Fig 2b). Furthermore, this is much more evident during the snowmelt period of high solar radiation. Compared to CTRL, the decrease of RMSE for both snow depth and upward shortwave radiation from ASS8 is smaller than that from ASS1, but there is still significant improvement from ASS8 particularly for upward shortwave radiation. This promotes our confidence in snow albedo assimilation using satellite images with a relatively low temporal resolution (8-day period).

### Offline simulations at the continental level

#### Upward shortwave radiation

**Fig 3:**
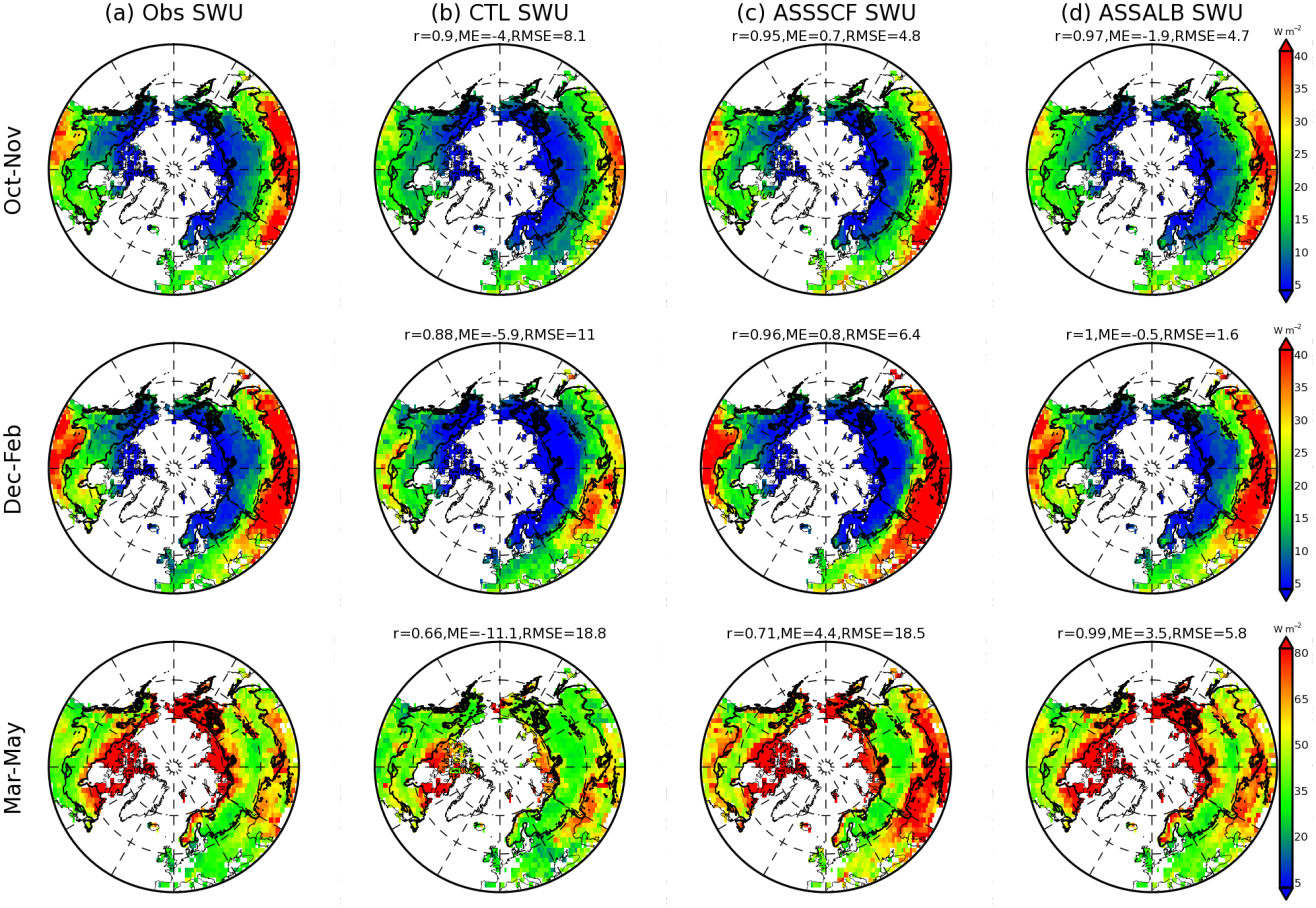
Mean upward shortwave radiation (SWU) (W m^-2^) during the period from 1998 to 2011 shown for observation, CTRL, ASSSCF and ASSALB for autumn (October and November), winter (December, January and February) and spring (March, April and May) seasons in the region north of 40 degrees. Observations are computed from CRUNCEP-derived incoming solar radiation multiplied by albedo from GlobAlbedo. CTRL represents the control simulation from ORCHIDEE, ASSALB denote the offline simulation that assimilates only GlobAlbedo SCF, and ASSALB is the offline simulation that assimilates both albedo and SCF from GlobAlbedo product. ‘r’ and RMSE represent the spatial correlation of simulation with observation and root mean square error, respectively.

The CTRL analysis reproduces well the observed spatial pattern of mean upward shortwave radiation for the area north of 40 degrees over the period 1998–2011 during the autumn (October and November), winter (December, January and February) and spring (March, April and May) (Fig 3). The observed upward shortwave radiation is approximated by multiplying GlobAlbedo albedo with CRUNCEP incoming shortwave radiation. The upward shortwave radiation over the boreal zone (thick black line in Fig 3) has a low value relative to other regions. On the one hand, this arises from the fact that received shortwave radiation is lower than that of the regions further south. On the other hand, this is due to the masking of snow albedo by widely distributed forests within the boreal zone (e.g. [39]). However, these masking effects have been overestimated by CTRL. Mean upward shortwave radiation over the boreal zone from CTRL during autumn, winter and spring is 11.3, 11.4 and 41.8 W m^-2^, respectively, which is lower than the equivalent observed figures of 14.0, 15.4 and 53.6 W m^-2^.

The ASSALB analysis (Fig 3d) shows significant improvements in simulating upward shortwave radiation with spatial correlations of 0.97~0.99, MEs of -0.5~3.5 W m^-2^ and RMSEs of 1.6~5.8 W m^-2^ when compared against observations in different seasons (Fig 3a). In contrast, for the CTRL case, the corresponding spatial correlations, MEs and the RMSEs are 0.66~0.90, -11.1~-4.0 W m^-2^ and 8.1~18.8 W m^-2^ respectively (Fig 3b). North of 40°N, the difference in upward shortwave radiation between CTRL and ASSALB can be as high as 7.5 W m^-2^ from October to May.

The ASSSCF analysis only assimilated with GlobAlbedo SCF indicates that the improvements in the simulation of upward shortwave radiation are mainly found in the north of 50 degrees but the simulations are degraded in the southern boundary especially during the spring (Fig 3c). Further analyses have shown that the region with degraded simulation has a lower SCF from ORCHIDEE than that from GlobAlbedo (data not shown). Since surface albedo is computed based on the combination of SCF, snow-covered and snow-free albedo (Eq 6) and snow-covered albedo is generally larger than the snow-free albedo, the over-estimation of upward shortwave radiation from ASSSCF in the southern boundary (Fig 3c) can then be mainly attributed to over-estimation of snow-covered albedo in ORCHIDEE. In addition, the improvements over the region north of 50 degrees are relatively small in ASSSCF compared to those from ASSALB (Fig 3d). This highlights the necessity of improving snow-covered albedo simulations in ORCHIDEE. One potential alternative is to utilize satellite-based albedo products (e.g. GlobAlbedo, MODIS) along with SCF maps to re-parameterize the default snow albedo scheme in ORCHIDEE (e.g. [14, 40]).

#### Snow water equivalent

**Fig 4:**
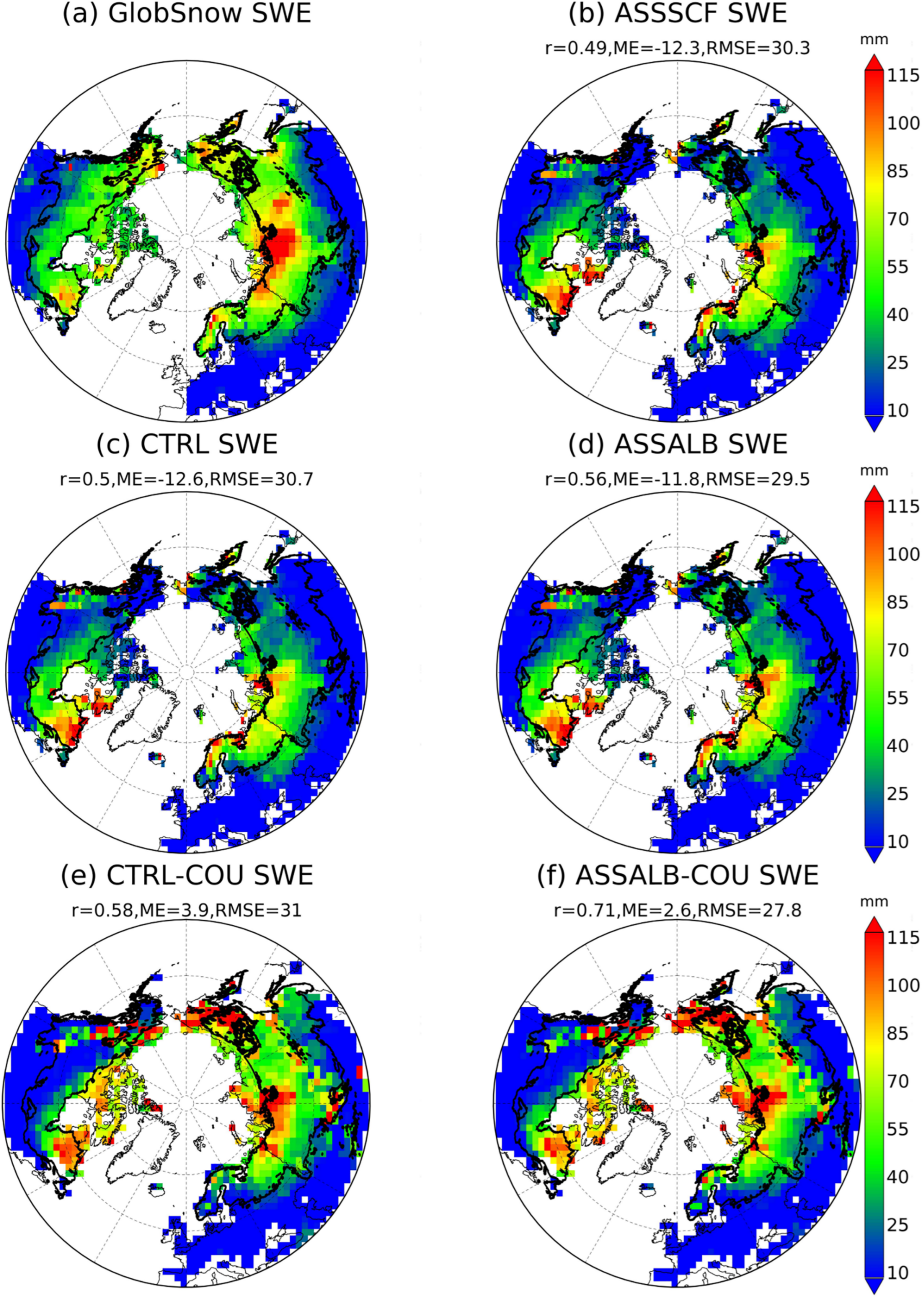
Spatial distribution of mean seasonal (October throughout May) snow water equivalent (SWE, mm) over the period 1998–2011 shown for GlobSnow, CTRL, ASSSCF, ASSALB, CTRL-COU and ASSALB-COU in the region north of 40 degrees. CTRL-COU represents the control simulation from the coupled ORCHIDEE-LMDZ model and ASSALB-COU denotes the coupled simulation that assimilates both albedo and SCF from GlobAlbedo product. The meanings of CTRL, ASSSCF and ASSALB are the same with those of Fig 3.

The gridded SWE dataset generated by combining satellite measurements with ground stations is used to assess the spatial distribution of simulated SWE. Fig 4 shows the spatial distribution of multi-year (1998–2011) mean seasonal (October throughout May) SWE from different simulations (CTRL, ASSSCF and ASSALB) over the region north of 40 degrees. The CTRL analysis roughly captures the spatial pattern of GlobSnow SWE with a spatial correlation, ME and RMSE of 0.50, -12.6 mm and 30.7 mm, respectively. However, there is still a high bias e.g. in the Siberia region, which may be ascribed to the inaccuracy of climate forcing because of sparse meteorological stations at high-latitude regions. Moreover, the bias in boreal zone may also be contributed by the inability of ORCHIDEE (i.e. the surface energy budget is based on the “big-leaf” approach) in modeling sub-canopy environments that may significantly affect snow accumulation and ablation in forested regions. This issue could be alleviated in the near future if the multi-layer snow scheme used in this study was introduced into a recently developed ORCHIDEE branch (called ORCHIDEE-CAN) in which a multi-layer land surface energy budget has been implemented [41].

**Fig 5:**
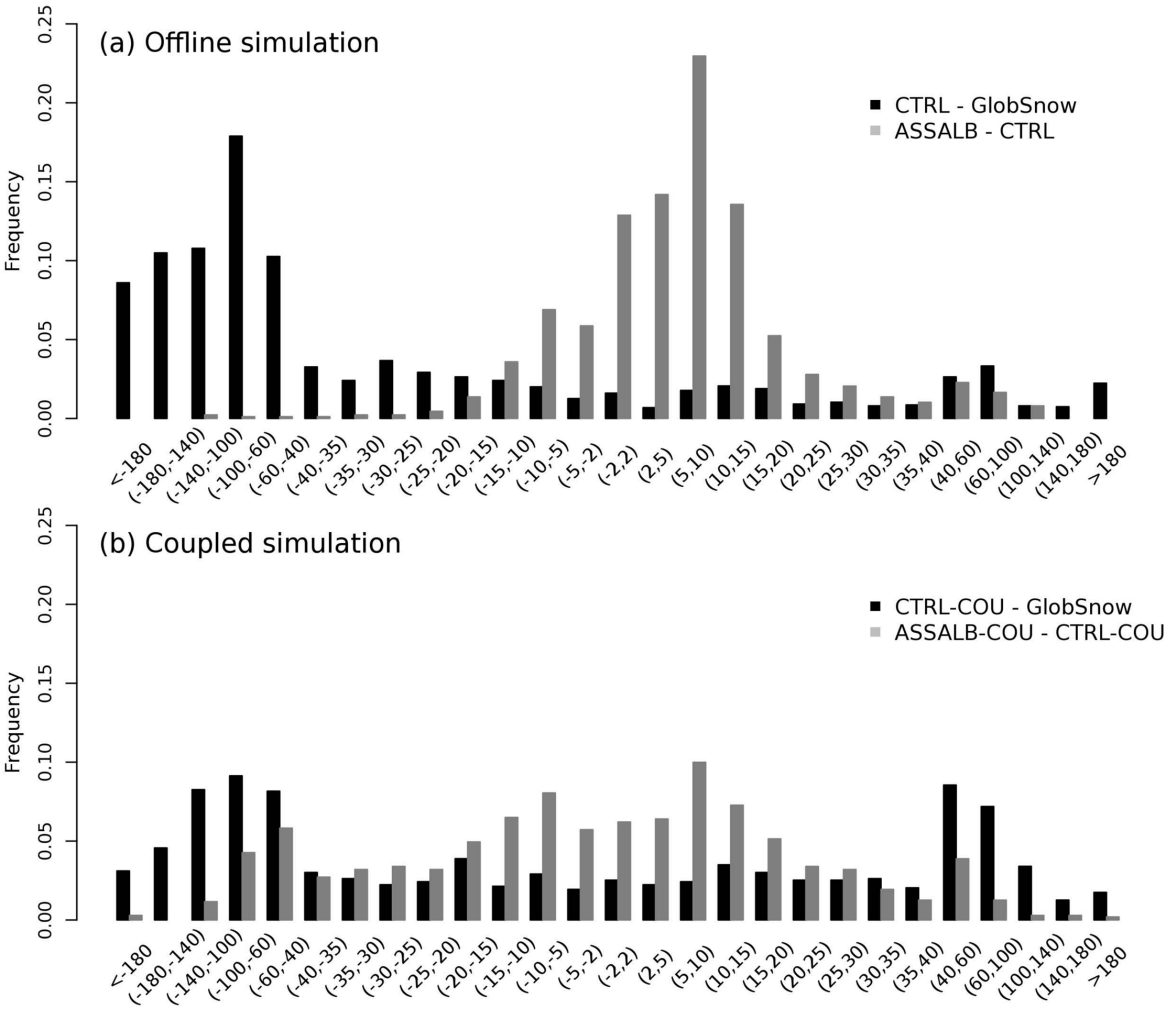
Histograms of relative change in mean seasonal (October throughout May) snow water equivalent (SWE, mm) over the period 1998–2011 in the offline simulation (a) and the coupled simulation (b).

Compared to CTRL (Fig 4c), minor improvements in SWE simulations are found in ASSSCF (ME = -12.3 mm; RMSE = 30.3 mm) (Fig 4b) and ASSALB (ME = -11.8 mm; RMSE = 29.5 mm) (Fig 4d). Compared to CTRL, the SWE changes in ASSSCF and ASSALB are relatively small (Fig 4b and 4d). This can be seen if the relative change of mean seasonal (October throughout May) SWE between ASSALB and CTRL (in percentage) were aggregated into different bins (Fig 5a). For example, more than 85% of pixels north of 40 degrees have a magnitude of relative SWE change less than 20% (gray bar). In this study, impacts of SCF assimilation on SWE are manifested through changing the surface albedo in ORCHIDEE, and this should be distinguished from previous studies that used SCF observations to directly update SWE in the model through establishing a physical link between SCF and SWE (e.g. [42, 43]).

### The impacts of snow albedo assimilation on air temperature and SWE in the coupled simulation

#### Surface albedo and upward shortwave radiation

**Fig 6:**
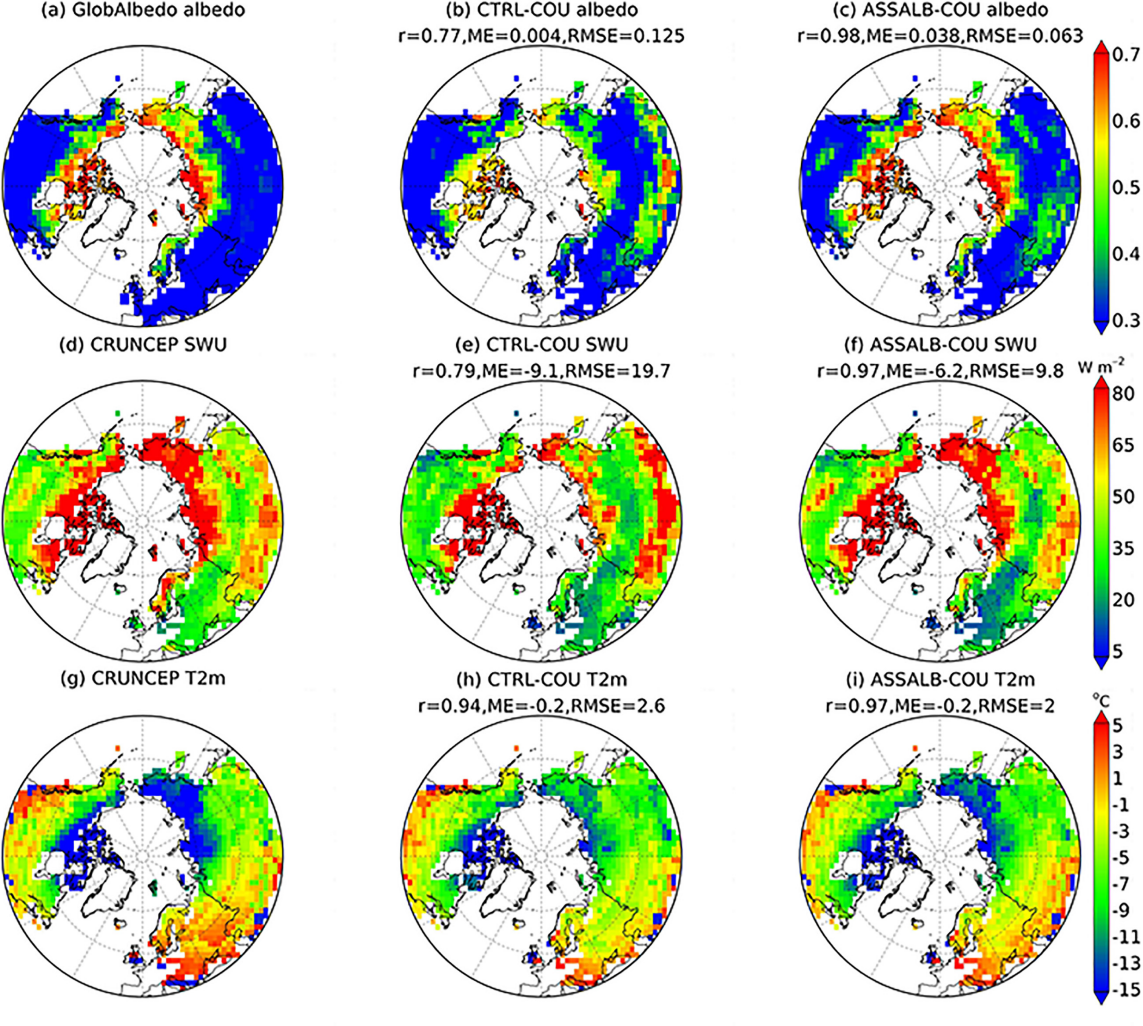
Mean spring (March, April and May) albedo (a-c), upward shortwave radiation (SWU) (d-f) and air temperature at 2 m (°C) (g-i) over the period 1998–2011 shown for observation, CTRL-COU and ASSALB-COU. The meanings of CTRL-COU and ASSALB-COU are the same with those of Fig 4.

If we use upward shortwave radiation that is calculated by multiplying GlobAlbedo-based albedo with CRUNCEP-based downward shortwave radiation as observation, ASSALB-COU has an improved performance in upward shortwave radiation simulation than CTRL-COU. For example, during the spring, ASSALB-COU (*r* = 0.97, ME = -6.2 W m^-2^, RMSE = 9.8 W m^-2^) has an enhanced spatial correlation and reduced errors compared to CTRL-COU (*r* = 0.79, ME = -9.1 W m^-2^, RMSE = 19.7 W m^-2^) (Fig 6e and 6f). This is also found during the autumn and winter (data not shown).

**Fig 7:**
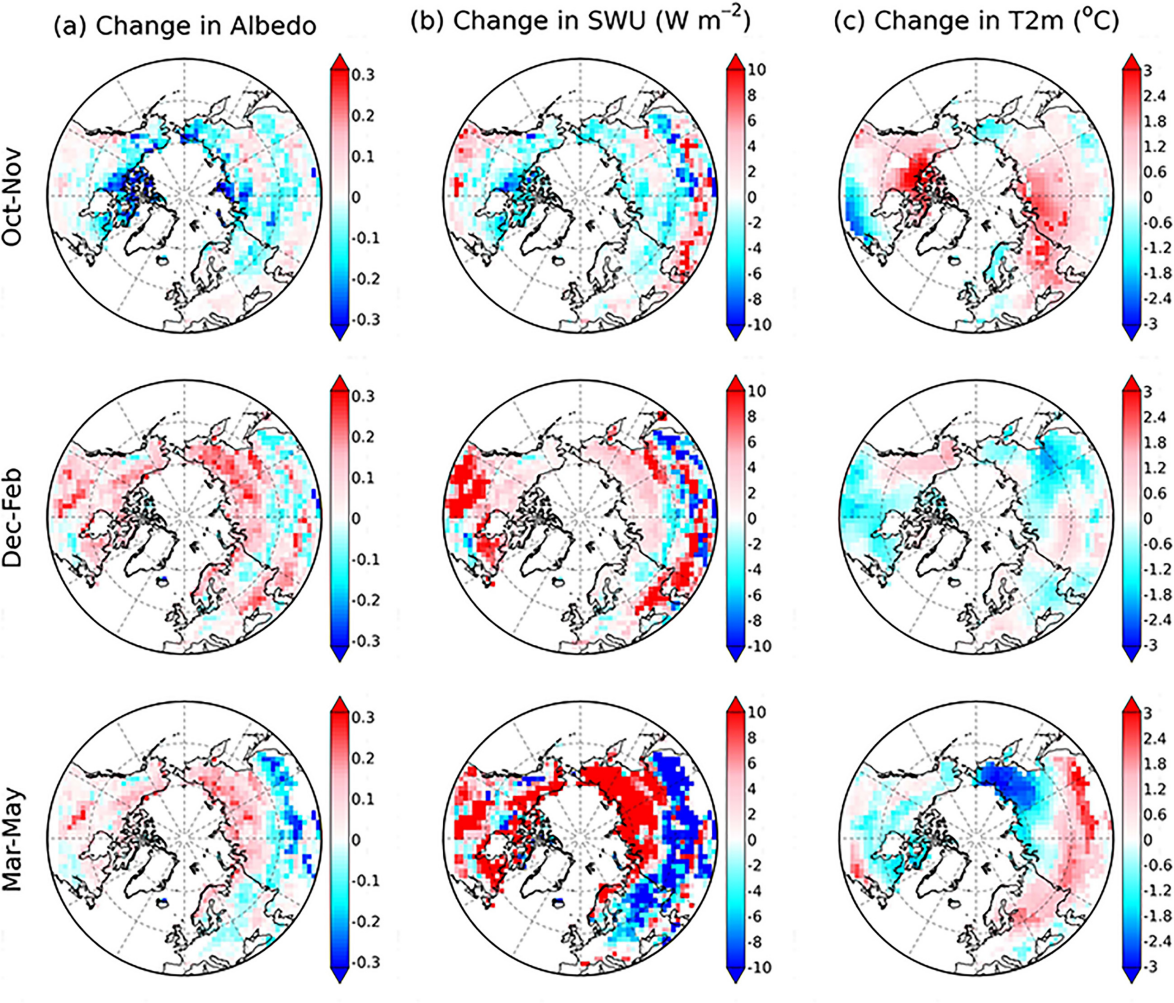
Spatial distribution of the difference between ASSALB-COU and CTRL-COU (ASSALB-COU minus CTRL-COU) in albedo (a), upward shortwave radiation (SWU) (b), air temperature at 2 m (c) shown for autumn (October and November), winter (December, January and February) and spring (March, April and May). The meanings of CTRL-COU and ASSALB-COU are the same with those of Fig 4.

The surface albedo and upward shortwave radiation from CTRL-COU are generally lower than those from ASSALB-COU during the winter (Fig 7a and 7b). By contrast, they are reversely found during the autumn. During the spring, compared to ASSALB-COU, CTRL-COU generally has a higher albedo and upward shortwave radiation in Eurasia south of 60 degrees but a lower albedo and upward shortwave radiation elsewhere (Fig 7a and 7b). We have noticed that in offline simulations the surface albedo and upward shortwave radiation from CTRL are generally lower than those from ASSALB in all seasons (e.g. Fig 3b). This is not surprising since the surface albedo is sensitive to climate forcing data (e.g. snowfall amount and its timing determine snow age and albedo) and the simulated climate from CTRL-COU is different from CRUNCEP used in CTRL (especially for total precipitation; data not shown).

Furthermore, the radiative forcing is calculated to quantify the instantaneous perturbation to Earth’s top-of-atmosphere energy balance induced by snow albedo assimilation in the coupled model. We define the radiative forcing as the difference of net radiation flux at the top of the atmosphere between ASSALB-COU and CTRL-COU over the region north of 40 degrees. The radiative forcing over the snow season (October-May) is estimated to be -1.82 W m^-2^, with a peak in spring of -2.50 W m^-2^. The magnitude of the spring value is much larger than that (0.45 W m^-2^) due to surface albedo changes induced by snow and ice from 1979 to 2008 in Northern Hemisphere [44] and comparable to that (2.83 W m^-2^) due to increase in CO_2_ concentration since 1750 [45]. This highlights the necessity of realistically representing snow albedo in the coupled simulation.

#### Air temperature

If air temperature from CRUNCEP was regarded as observations, spring temperature simulation has been improved in the coupled model due to snow albedo assimilation (Fig 6h and 6i). For example, ASSALB-COU (RMSE = 2°C) has less errors than CTRL-COU (RMSE = 2.6°C) over pixels with GlobAlbedo SCF larger than 10%. This is notably found in the eastern Siberia region where a negative bias of simulated albedo in CTRL-COU (Fig 6b) has been largely removed in ASSALB-COU (Fig 6c). We could also see an improvement in autumn temperature simulation due to snow albedo assimilation (data not shown), although which is not as strong as that in spring temperature. But there is little benefit for winter temperature simulation (data not shown).

To further understand the seasonal discrepancy in influences of snow albedo assimilation on air temperature, we calculate spatial sensitivity of T_2m_ change to albedo change (the difference between ASSALB-COU and CTRL-COU), which is defined as the linear coefficient between T_2m_ change and albedo change across pixels with GlobAlbedo SCF larger than 10%. We find that the absolute magnitude of spatial albedo sensitivity of temperature is higher during the spring (-0.85°C per 10% change in albedo, *r* = 0.70, *p* < 0.01) than during the autumn (-0.44°C per 10% change in albedo, *r* = 0.42, *p* < 0.01) and winter (-0.17°C per 10% change in albedo, *r* = 0.24, *p* < 0.01), implying that snow albedo feedback is much stronger during the spring than during the autumn and winter. Our results suggest that realistic representation of surface albedo is more important in the simulation of air temperature during the spring with high shortwave radiation than during the winter with minimum shortwave radiation.

#### Snow water equivalent

CTRL-COU has a better performance in capturing the spatial pattern of mean seasonal (October throughout May) GlobSnow SWE than CTRL in the boreal zone of Eurasia but not in North America (Fig 4a, 4c and 4e). The mean spatial error of seasonal SWE switches from a relatively large under-estimation (-12.6 mm) from CTRL (Fig 4c) to a slight over-estimation (3.9 mm) from CTRL-COU (Fig 4e). This is partly a result of the fact that CTRL-COU has a higher amount of precipitation than CRUNCEP. In consistent with offline simulations, we could also identify an improvement in the simulation of spatial SWE in the coupled model after snow albedo assimilation (Fig 4e and 4f). For example, ASSALB-COU (*r* = 0.71, ME = 2.6 mm, RMSE = 27.8 mm) has an enhanced spatial correlation and reduced errors compared to CTRL-COU (*r* = 0.58, ME = 3.9 mm, RMSE = 31.0 mm).

Since the feedback of albedo on surface climate (e.g. temperature and precipitation) is allowed in the coupled mode, the change in mean seasonal SWE (the percentage difference between CTRL-COU and ASSALB-COU) due to snow albedo assimilation (Fig 5b, gray bar) is found to be more pronounced in the coupled simulation than its counterpart in the offline simulation (Fig 5a, gray bar). For example, nearly 30% of all pixels north of 40 degrees have the magnitude of SWE change greater than 30%, which is higher than 8% from the offline simulations.

## Conclusions

This study implements a direct insertion methodology to assimilate snow albedo from observations in the land surface model ORCHIDEE. In both offline and coupled simulations, our study demonstrates the feasibility and utility of snow albedo assimilation from satellite images with a relatively low temporal resolution (8 days in our case), which is relatively easy to obtain compared to the high temporal resolution data (e.g. daily). The assimilation of only snow cover fraction in the offline model does not always lead to an improved simulation in surface albedo, since which may also be dependent upon the simulation accuracy of snow-covered albedo in ORCHIDEE. It highlights the necessity of improving the current snow albedo scheme for snow-covered albedo on different PFTs in ORCHIDEE. This can be achieved by optimizing PFT-specific parameters (albedo decay rate and albedo for old snow) using GlobAlbedo product. More importantly, this study is the first attempt to quantify impacts of snow albedo assimilation in the coupled model. We have demonstrated that a realistic representation of surface albedo during the snow season can partly remove the bias in temperature simulations, which is particularly found during the spring when the strength of snow albedo feedback is the largest. This has significant implications for our ongoing work. More specifically, it will help us to re-evaluate the strength of snow albedo feedback during the spring and quantify the contribution of snow albedo feedback to recent changes in spring temperatures over the Northern Hemisphere. Moreover, it will also assist us in testing the proposed mechanism linking increasing autumn snow cover to the recent widespread winter cooling in the boreal region [46].

A potential criticism of this study is that we do not take into account any potential errors in the satellite dataset and albedo from GlobAlbedo product is assumed to be accurate with respect to model simulations. In reality, significant artifacts exist at high latitudes (north of 70°) in the GlobAlbedo product because of problems with snow detection, although a preliminary validation of the GlobAlbedo product displayed a generally good agreement with the MODIS albedo product on the global scale [47]. But this issue is not specific to the GlobAlbedo product. For example, an inter-comparison of nine satellite-derived datasets has shown that albedo differences are large for early spring (February-April) in the northern hemisphere and also for the winter season particularly at high latitudes [30]. Therefore, more work is necessary in order to understand and resolve inconsistencies amongst these datasets, especially during winter and spring seasons (e.g. [30, 48]).
